# Characteristics of sleep-disordered breathing in children with down syndrome - A comparison with typically developing children

**DOI:** 10.1016/j.sleepx.2022.100045

**Published:** 2022-04-07

**Authors:** Hiroyuki Sawatari, Anita Rahmawati, Nobuko Moriyama, Kanae Fujita, Tomoko Ohkusa, Tomoko Nao, Nobuko Hashiguchi, Mari Nishizaka, Shin-ichi Ando, Akiko Chishaki

**Affiliations:** a Department of Perioperative and Critical Care Management, Graduate School of Biomedical and Health Sciences, Hiroshima University, Hiroshima, Japan; b Department of Biomedical Sciences, Faculty of Medicine, Universitas Padjadjaran, Bandung, West Java, Indonesia; c Department of Health Sciences, Kyushu University Graduate School of Medical Sciences, Fukuoka, Japan; d Faculty of Health Sciences, Department of Nursing, Ube Frontier University, Yamaguchi, Japan; e Kirameki Project Carrier Support Center, Kyushu University Hospital, Fukuoka, Japan; f Sleep Apnea Center, Kyushu University Hospital, Fukuoka, Japan; g Fukuoka Dental College Hospital and Fukuoka Nursing College, Graduate School of Nursing, Fukuoka, Japan

**Keywords:** Down syndrome, Sleep-disordered breathing, Children, Sleep posture, Parental reports

## Abstract

**Background:**

Compared with typically developing control children (CC), children with Down syndrome (DS) frequently exhibit sleep-disordered breathing (SDB) and unusual sleep postures (USPs). No studies have directly compared SDB-related signs and symptoms, SDB-related parameters, and USPs between children with DS and CC. This study aimed to evaluate the prevalences of SDB and USPs in children with DS and CC.

**Methods:**

We analyzed SDB-related parameters measured via overnight pulse oximetry and questionnaires administered to parents on SDB-related signs and symptoms, including sleeping postures. Estimated SDB was defined as a 3% oxygen desaturation index (ODI) ≥5 dips/h.

**Results:**

Fifty-one children with DS (4–5 years: N = 12, 6–10 years: N = 23, 11–15 years: N = 16) and sixty-three CC (4–5 years: N = 18, 6–10 years: N = 27, 11–15 years: N = 18) were included. The prevalence of estimated SDB and observed USPs was higher in children with DS than in CC (p < 0.0001). Among children aged 11–15 years old, but not those aged 4–5 and 6–10 years old, frequency of arousal and apnea (p = 0.045 and p = 0.01, respectively) were higher in children with DS than in CC. Multivariate analyses showed that DS was associated with SDB-related signs and symptoms, estimated SDB, 3% ODI, average oxygen saturation (SpO_2_), and nadir SpO_2_, while USPs were associated only with higher values of SpO_2_ <90%.

**Conclusions:**

Estimated SDB tended to increase in children with DS but decreased in CC with growth. USPs were more frequent in children with DS than in CC, especially in older children. USPs might indicate severe hypoxemia due to SDB in DS.

## Highlights

• The difference of prevalence of SDB/USPs between DS and CC was evaluated.
• The prevalence of SDB increased with growth in the DS but not in the CC.
• USPs, taking to escape from suffocation by SDB, were frequently observed in older DS.

## 1 Introduction

Down syndrome (DS) is a common inherited chromosomal disorder, which is estimated to occur in 1/650 to 1/1000 live births [1,2]. Children with DS are at an increased risk of developing sleep-disordered breathing (SDB) than typically developing children [3,4]. SDB in DS results mainly from hypotonia, macroglossia, and midface hypoplasia [5]. Progressive obesity is another risk factor in adolescents with DS [6]. Children with DS also have an increased risk for congenital heart disease (CHD) and pulmonary hypertension, which are potentially associated with disrupted sleep [6,7]. Since the presence of SDB in children could be a risk factor for attention difficulty, disturbance of normal intellectual development, and exacerbation of cardiovascular diseases, early detection and treatment of SDB should be mandatory in children, even those without DS [8,9].

The American Academy of Pediatrics recommends a sleep study to screen for SDB in children with DS, even at early ages. However, a sleep study using type-1 polysomnography (PSG) has several disadvantages, including its high cost and the difficulty in performing the test with many sensors, especially in children with DS who also have intellectual disability [10,11]. Initial screening with pulse oximetry could be an alternative tool for cases in which a diagnostic multichannel PSG study is difficult to perform, and it might provide patients early access to essential therapy for SDB [12,13].

A previous study reported that unusual sleep postures (USPs), such as sitting and leaning forward, were frequently observed in patients with DS [14]. Positional therapies are known to be effective in obstructive sleep apnea (OSA) [15]. If the USPs in children were self-defending behaviors to prevent airway collapse during sleep, then these could be considered supportive signs for the existence of SDB. Since parent-child co-sleeping is common in Asian countries, we hypothesized that we could collect reliable data from parents regarding their children's sleep characteristics or postures that might help in the early detection of SDB [16].

Accordingly, the objectives of the current case–control study were as follows: 1) to assess the differences in SDB-related parameters and SDB-related signs and symptoms, including USPs, between children with DS and typically developing control children (CC) in different age groups, and 2) to examine the relationships between SDB-related parameters and USPs in children with DS and CC.

## 2 Methods

### 2.1 Participants

We recruited children with DS who were members of the Japan Down Syndrome Society in Yamaguchi and Fukuoka Prefectures. An explanatory letter, questionnaires, and a pulse oximeter were sent via post. After completing the questionnaire and overnight pulse oximetry measurements, the parents returned them to the researchers in prepaid envelopes. Children without DS (ie, CC) who had no underlying diseases were randomly selected from several elementary and junior high schools in the same prefectures as the children with DS, and these included the siblings of children with DS. This research was conducted between 2015 and 2018, with the understanding and written informed consent of each participant and their parents. The study was approved by the Ethics Committee of Kyushu University Graduate School of Medical Sciences and Ube Frontier University. The study procedures followed were in accordance with the Declaration of Helsinki.

### 2.2 Overnight pulse oximetry

Children with DS and CC underwent overnight pulse oximetry measurements (PULSOX-Me300, Konica Minolta Sensing, Inc., Tokyo, Japan) for 2 or 3 consecutive nights at home. In addition to providing the parents written instructions and illustrations about performing overnight pulse oximetry measurements, a mini-seminar was held to explain the use of the device. A pulse oximeter was attached to the study participants before sleeping at home. To avoid the first-night effect, the recording results from the first night were discarded. We used the 3% oxygen desaturation index (ODI), representing the number of ≥3% desaturation events per hour of recording time, as a marker for the frequency of hypoxemia. The criteria for SDB in children for evaluating the presence of SDB are still under debate [4]. We set the cutoff value for the definition of SDB as 3% ODI ≥5 dips/h, which is same definition as that in adults. The average arterial oxygen saturation level (average SpO_2_), ratio of arterial oxygen saturation less than 90% (SpO_2_ <90%), and nadir values of arterial oxygen saturation levels (nadir SpO_2_) were also used. All recorded data were manually double-checked by a certified sleep technician and a medical doctor, both of whom were blinded to the participants’ characteristics, to adopt only artifact-free recording periods, using a data analysis software (DS-Me version. 2.10; Minolta, Tokyo, Japan).

### 2.3 Questionnaires

**Fig. 1:**
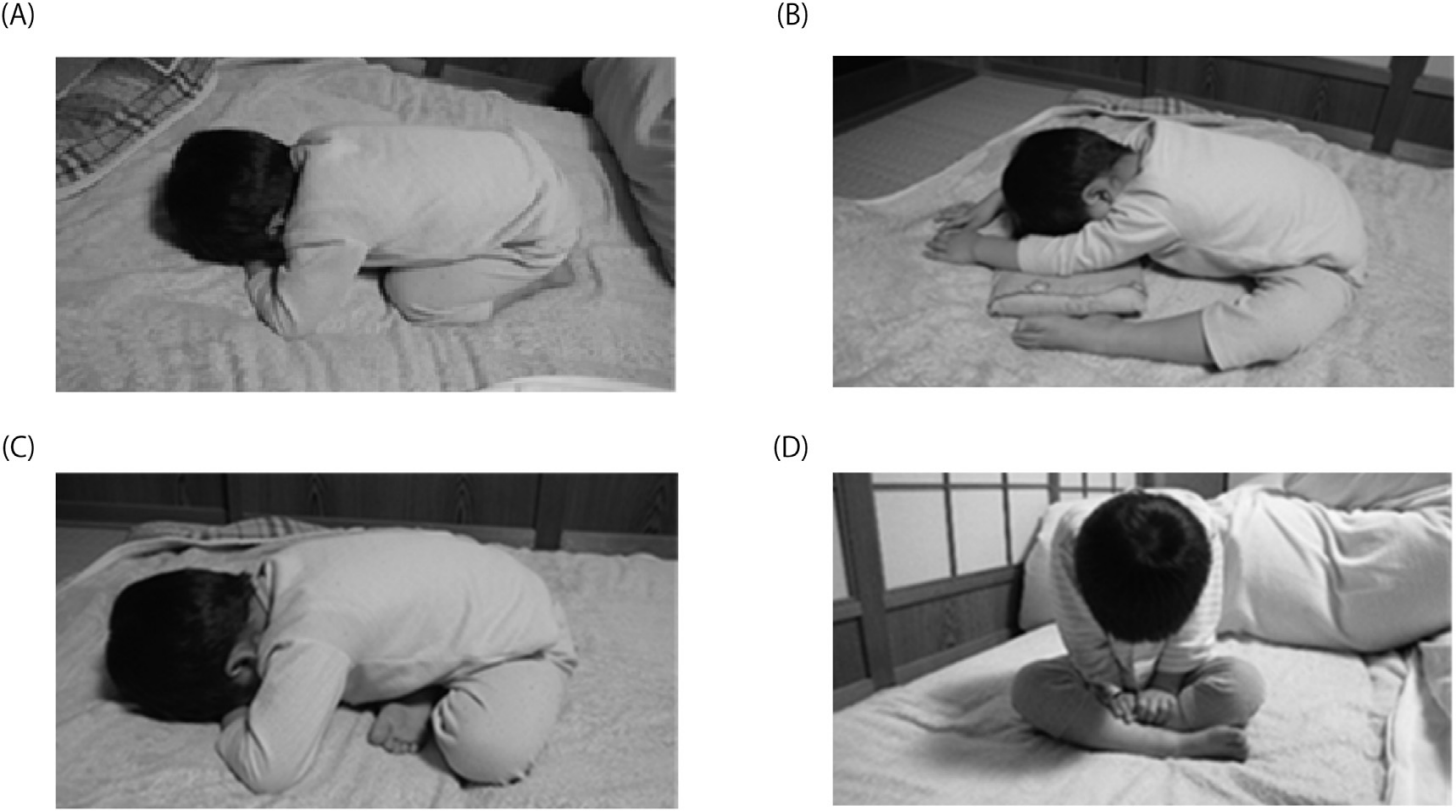
Unusual sleep postures. (A) Leaning forward with legs back. (B) Leaning forward with legs front. (C) Leaning forward with legs cross. (D) Sitting.

The parents completed a questionnaire on age, sex, body weight, body height, presence of CHD, and SDB-related signs and symptoms, such as witnessed midnight arousal, snoring, apnea, and daytime napping [17,18]. The Japanese version of the Epworth Sleepiness Scale (ESS) was also answered by the parents, and a higher ESS value indicated that the person had more severe daytime sleepiness [10,19]. Witnessed apnea was defined as a complete pause of breathing followed by restarting of breathing with rapid gasping for air [10]. The questionnaire for SDB-related signs and symptoms was answered by selecting one of the four following choices: “Frequently” (5–7 nights/week), “Sometimes” (1–4 nights/week), “Never” (0 night/week), or “Unknown.” When the parents answered a question as “Frequently” or “Sometimes,” we regarded the answers as “positive” or “yes.” We also regarded the data as “Missing” when the parents answered a question as “Unknown,” and thus, the denominator of each variable for calculating the percentage was different from the others. Because parent-child co-sleeping is common, especially in children with comorbid conditions in Japan, habitual sleep postures and subjective sleeping time are evaluated on the basis of parental observation [16]. Sleep postures were categorized as usual (supine and lateral), prone, and unusual (leaning forward with legs backward, leaning forward with legs forward, leaning forward with legs crossed, and sitting) (Fig. 1). Sleep postures were classified as usual postures in the absence of unusual or prone postures. USPs were defined as the presence of one or more sitting or leaning forward postures (legs backward, forward, or crossed), and a prone posture was defined as a prone posture without USPs [10]. The subjective sleeping time was estimated as the sleeping time based on data from the questionnaires for overnight pulse oximetry measurements.

### 2.4 Statistical analysis

STATA version 15.1 (Stata-Corp, TX, USA) was used for statistical analyses. Statistical tests were two-sided, with a significance level set at p < 0.05. Continuous variables were compared using Student's t-test or the Mann–Whitney U-test after performing the Shapiro–Wilk test. Fisher's exact test was used to compare discrete variables. Means and standard deviations or medians and interquartile ranges were the main descriptive statistics for continuous variables, while frequencies and percentages were used to report categorical variables. The differences in the values of the variables were compared between the DS and CC groups. Additionally, comparisons were made by dividing all children into the following three age groups: 4–5 years, 6–10 years, and 11–15 years. This age grouping was based on the findings of a previous study on children with DS and CC which showed that younger children's sleep is developmentally different from teenagers' sleep [20]. Regarding the assessment of factors for SDB-related signs and symptoms and overnight oximetry-related parameters, we adequately assessed these relationships using regression analysis, and the results were shown as univariate analysis (Model 1) and multivariate analysis (Model 2). Variables with p < 0.10 in the univariate analysis were included in the multivariate analysis. Odds ratios with their corresponding 95% confidence intervals were reported for the logistic regression analysis, while standardized β was reported for the linear regression analysis.

## 3 Results

### 3.1 Overall comparisons between children with DS and CC

We evaluated the data from 51 children with DS and 63 CC with acceptable overnight pulse oximetry recordings. Children with DS had a lower body height than that of CC (p = 0.006) (Table 1). The body mass index (BMI) was not significantly different between the two groups. Children with DS had more frequent CHD than that of CC (p < 0.0001). SDB-related signs and symptoms, such as witnessed midnight arousal, snoring, witnessed apnea, and daytime napping, were more frequently observed in children with DS than in CC (p = 0.02, p = 0.007, p < 0.0001, and p = 0.003, respectively). Although the subjective sleeping time did not differ between children with DS and CC, the severity of daytime sleepiness indexed using the ESS was significantly higher in children with DS (p = 0.0001). Prevalence of estimated SDB (ie, 3% ODI ≥5 dips/h) was higher in children with DS (p < 0.0001), and the SDB-related parameters measured using pulse oximetry were worse in children with DS (3% ODI, p < 0.0001; average SpO_2_, p = 0.004; SpO_2_ <90%, p = 0.0004; nadir SpO_2_, p = 0.0003). Regarding sleep postures, 52.9% of children with DS had USPs, while only 9.5% of CC had USPs (p < 0.0001). Specifically, the sitting sleep posture was found only in children with DS.

**Table 1:** Clinical characteristics.

	DS	CC	P-value
**Number**	51	63	–
**Age,** years	8.0 (6.0–12.0)	7.0 (5.0–12.0)	0.62
**Male**, N (%)	26 (51.0)	34 (54.0)	0.85
**Physique**			
Body weight, kg	20.0 (16.6–38.0)	23.6 (18.7–39.0)	0.07
Body height, cm	112.6 (102.0–136.0)	122.0 (110.0–146.0)	0.006
BMI, kg/m^2^	16.3 (15.3–19.2)	16.4 (14.8–18.0)	0.38
**CHD**, N (%)	35 (68.6)	0 (0.0)	<0.0001
**Questionnaire**			
Arousal, N (%)	29 (56.9)	22 (34.9)	0.02
Snoring, N (%)	29 (58.0)^a^	18 (31.6)^a^	0.007
Apnea, N (%)	10 (27.8)^a^	0 (0.0)^a^	<0.0001
Napping, N (%)	16 (34.0)^a^	6 (9.7)^a^	0.003
ESS, points	2.0 (1.0–5.0)	1.0 (0.0–2.0)	0.0001
Subjective sleeping time, hour	8.5 (6.9–9.3)	8.9 (7.7–9.4)	0.17
**Overnight oximetry**			
Estimated SDB, N (%)	26 (51.0)	9 (14.3)	<0.0001
3%ODI, dips/hour	5.4 (3.2–7.6)	1.6 (0.8–2.6)	<0.0001
Averaged SpO_2_, %	96.5 ± 0.8	97.1 ± 0.9	0.004
SpO_2_<90%, %	5.0 (0.0–10.0)	1.0 (0.0–3.0)	0.0004
Nadir SpO_2_, %	82.3 (79.2–87.0)	88.0 (83.0–90.0)	0.0003
**Sleep postures**			
Unusual, N (%)	27 (52.9)	6 (9.5)	<0.0001
Leaning forward	25 (49.0)	6 (9.5)	<0.0001
Sitting	9 (19.2)^a^	0 (0.0)^a^	<0.0001
Prone, N (%)	13 (36.1)^a^	23 (37.7)^a^	0.22

DS: Down syndrome, CC: Control children, N: Number, BMI: Body mass index, CHD: Congenital heart diseases, SDB: Sleep-disordered breathing, ESS: Epworth sleepiness scale, ODI: Oxygen desaturation index.

a Since each answer had missing data (ie, answered as “Unknown”), the denominator of the field differed from the others.

### 3.2 Differences between children with DS and CC in various age groups

Children with DS and CC were stratified into three age groups according to the growing process and different school life systems: 4–5 years, 6–10 years, and 11–15 years. Using these groups, 51 children with DS were divided into 4–5 years (N = 12), 6–10 years (N = 23), and 11–15 years (N = 16), and 63 CC were also divided into 4–5 years (N = 18), 6–10 years (N = 27), and 11–15 years (N = 18) (Table 2) [20]. Body height was significantly lower in children with DS than in CC in all three age groups, and body weight was lower in children with DS than in CC in the age groups 4–5 years and 6–10 years, but the body weights of the children with DS and CC were similar in the age group 11–15 years. Thus, BMI was not significantly different between the children with DS and CC in the age groups 4–5 years and 6–10 years, but the BMI in the children with DS was significantly higher than that in CC in the age group 11–15 years (p = 0.03). Furthermore, a tendency toward obesity was observed after the end of the growth period of body height in children with DS.

**Table 2:** Differences between children with Down syndrome and typically developing children.

	4–5 years			6–10 years			11–15 years		
	DS	CC	P-value	DS	CC	P-value	DS	CC	P-value
**Number**	12	18	–	23	27	–	16	18	–
**Age**, years	4.5 (4.0–5.0)	5.0 (5.0–5.0)	0.06	7.7 ± 1.3	7.3 ± 1.4	0.34	13.0 ± 1.2	13.3 ± 1.1	0.41
**Male**, N (%)	5 (41.7)	8 (44.4)	1.00	12 (52.2)	16 (59.3)	0.78	9 (56.3)	10 (55.6)	1.00
**Physique**									
Body weight, kg	14.1 ± 1.9	17.7 ± 3.1	0.001	19.7 (18.0–22.0)	23.0 (21.0–27.6)	0.003	41.6 ± 8.9	45.8 ± 6.4	0.12
Body height, cm	97.0 ± 7.5	107.5 ± 7.7	0.001	111.8 ± 7.6	123.5 ± 10.4	<0.0001	140.3 ± 6.9	155.2 ± 8.1	<0.0001
BMI, kg/m^2^	15.0 ± 1.3	15.3 ± 1.4	0.65	15.8 (15.3–16.9)	16.0 (14.8–17.2)	0.48	21.0 (18.9–22.3)	18.8 (17.7–19.7)	0.03
**CHD,** N (%)	10 (83.3)	0 (0.0)	<0.0001	17 (73.9)	0 (0.0)	<0.0001	8 (50.0)	0 (0.0)	0.001
**Questionnaire**									
Arousal, N (%)	6 (50.0)	7 (38.9)	0.71	11 (47.8)	8 (29.6)	0.25	12 (75.0)	7 (38.9)	0.045
Snoring, N (%)	6 (50.0)	6 (33.3)	0.46	13 (56.5)	7 (29.2)	0.08	10 (66.7)	5 (33.3)	0.14
Apnea, N (%)	2 (18.2)^a^	0 (0.0)^a^	0.18	3 (21.4)^a^	0 (0.0)^a^	0.06	5 (45.5)^a^	0 (0.0)^a^	0.01
Napping, N (%)	4 (36.4)^a^	2 (11.1)	0.16	6 (28.6)^a^	1 (3.9)^a^	0.04	6 (40.0)^a^	3 (16.7)	0.24
ESS, points	3.0 (1.0–5.0)	1.0 (0.0–3.0)	0.04	1.0 (0.0–3.0)	0.0 (0.0–1.0)	0.051	2.0 (2.0–5.0)	1.0 (0.0–2.0)	0.005
Subjective sleeping time, hour	9.1 (8.5–10.1)	9.0 (7.1–10.2)	0.83	8.6 (7.7–9.1)	9.0 (8.5–9.3)	0.23	6.8 (6.2–7.4)	8.4 (7.7–9.4)	0.02
**Overnight oximetry**									
Estimated SDB, N (%)	4 (33.3)	6 (33.3)	1.00	12 (52.2)	3 (11.1)	0.002	10 (62.5)	0 (0.0)	<0.0001
3%ODI, dips/hour	4.5 (3.4–8.3)	2.5 (1.8–5.8)	0.052	5.5 (3.1–7.6)	1.3 (0.8–1.6)	<0.0001	5.6 (3.3–9.5)	1.2 (0.5–2.0)	0.0001
Averaged SpO_2_, %	96.6 ± 0.5	97.2 ± 1.0	0.12	96.5 (96.2–97.2)	97.0 (96.3–97.7)	0.10	96.6 ± 0.8	97.2 ± 0.6	0.03
SpO_2_<90%, %	6.0 (3.0–10.0)	3.0 (2.0–16.0)	0.41	5.0 (2.0–14.0)	0.0 (0.0–2.0)	0.005	3.0 (2.0–20.0)	1.0 (0.0–2.0)	0.008
Nadir SpO_2_, %	81.2 (79.0–83.8)	83.2 (80.5–89.0)	0.18	82.3 (79.0–87.0)	89.0 (84.0–91.0)	0.004	85.7 (81.2–88.1)	88.0 (858–93.0)	0.02
**Sleep postures**									
Unusual, N (%)	7 (58.3)	3 (16.7)	0.045	10 (43.5)	3 (11.1)	0.02	10 (62.5)	0 (0.0)	<0.0001
Leaning forward	7 (58.3)	3 (16.7)	0.045	8 (34.8)	3 (11.1)	0.08	10 (62.5)	0 (0.0)	<0.0001
Sitting	2 (16.7)	0 (0.0)	0.15	3 (15.8)^a^	0 (0.0)^a^	0.07	4 (25.0)	0 (0.0)^a^	0.10
Prone, N (%)	2 (16.7)	5 (27.8)	0.67	6 (26.1)	13 (50.0)^a^	0.40	5 (31.3)	5 (29.4)^a^	1.00

DS: Down syndrome, CC: Control children, N: Number, BMI: Body mass index, CHD: Congenital heart diseases, SDB: Sleep-disordered breathing, ESS: Epworth sleepiness scale, ODI: Oxygen desaturation index.

a Since each answer had missing data (ie, answered as “Unknown”), the denominator of the field differed from the others.

Among the SDB-related signs and symptoms, significant differences in witnessed midnight arousal and apnea were not observed between children with DS and CC in the age groups 4–5 years and 6–10 years, but such signs and symptoms were more frequently observed in children with DS than in CC in the age group 11–15 years (75.0 vs. 38.9%, p = 0.045 and 45.5 vs. 0.0%, p = 0.01, respectively). Moreover, overnight pulse oximetry parameters were not significantly different between children with DS and CC in the age group 4–5 years. However, in the age groups 6–10 years and 11–15 years, the ratios of estimated SDB were significantly higher, and other pulse oximetry parameters were significantly worse in children with DS than in CC.

USPs were significantly more frequent in children with DS than in CC in all age groups. USPs were also observed in CC, but their frequency declined along with growth. Children with DS showed no such decline in USPs. Sitting position was observed in 15–25% of children with DS, while none of the CC slept in the sitting position, regardless of age.

Next, we compared differences between pre-pubertal (4–10 years old) and pubertal (11–15 years old) periods in children with DS and CC, separately (Supplementary Table 1). In the CC, the rate of estimated SDB in pre-pubertal children was significantly higher than that in pubertal children (20.0 vs. 0.0%, p = 0.04). In the children with DS, on the other hand, the significant decreases of SDB-related parameters and USPs noted in CC were not observed, and the reported arousal tended to worsen in the pubertal period. SDB related parameters and USPs were consistently high values in both pre-pubertal and pubertal periods in children with DS.

### 3.3 Factors associated with the presence of SDB

Table 3 shows the factors associated with the SDB-related signs and symptoms, using regression analyses. In the univariate analysis, the presence of DS was significantly associated with witnessed midnight arousal, snoring, daytime napping, and higher ESS values, whereas in the multivariate analysis, the presence of DS was significantly associated with snoring, daytime napping, and higher ESS values (p = 0.007, p = 0.02, and p = 0.001, respectively). Other factors such as male sex, age, BMI, and the presence of USPs were not associated with the SDB-related signs and symptoms in the multivariate analysis.

**Table 3:** Factors for presence of symptoms of sleep-disordered breathing.

Model 1	Arousal		Snoring		Apnea		Napping		ESS	
	OR (95% CI)	P-value	OR (95% CI)	P-value	OR (95% CI)	P-value	OR (95% CI)	P-value	β	P-value
DS	2.46 (1.15–5.25)	0.02	2.99 (1.36–6.61)	0.007	N/A	–	4.82 (1.71–13.57)	0.003	0.35	<0.0001
Male	0.77 (0.37–1.61)	0.49	1.06 (0.49–2.28)	0.88	0.22 (0.04–1.10)	0.07	1.17 (0.46–3.00)	0.74	0.02	0.84
Age	1.04 (0.94–1.16)	0.46	1.05 (0.94–1.17)	0.40	1.14 (0.95–1.36)	0.17	1.07 (0.94–1.22)	0.92	0.03	0.76
BMI	1.12 (0.98–1.29)	0.10	0.98 (0.86–1.12)	0.75	1.16 (0.91–1.47)	0.24	1.10 (0.95–1.28)	0.21	0.06	0.56
Estimated SDB	3.49 (1.51–8.06)	0.003	2.65 (1.16–6.08)	0.02	9.83 (2.25–42.88)	0.002	1.04 (0.38–2.83)	0.94	0.10	0.27
Unusual sleep posture	2.48 (1.08–5.69)	0.03	1.19 (0.52–2.72)	0.69	2.20 (0.55–8.70)	0.26	2.79 (1.05–7.41)	0.04	0.17	0.07
Model 2	OR (95% CI)	P-value	OR (95% CI)	P-value	OR (95% CI)	P-value	OR (95% CI)	P-value	β	P-value
DS	1.93 (0.83–4.52)	0.13	2.99 (1.36–6.61)	0.007	–	–	4.09 (1.32–12.70)	0.02	0.35	0.001
Male	–	–	–	–	0.22 (0.04–1.10)	0.07	–	–	–	–
Age	–	–	–	–	–	–	–	–	–	–
BMI	–	–	–	–	–	–	–	–	–	–
Unusual sleep posture	1.79 (0.70–4.55)	0.23	–	–	–	–	1.49 (0.50–4.44)	0.48	0.006	0.95

Model 1: Clued, Model 2: Multivariate analysis. Hyphen indicates variable which did not include the multivariate analysis.

DS: Down syndrome, BMI: Body mass index, SDB: Sleep-disordered breathing, ESS: Epworth sleepiness scale.

Regarding the overnight oximetry data, the presence of DS was also significantly associated with a higher prevalence of estimated SDB, higher values of 3% ODI, and lower values of average SpO_2_ and nadir SpO_2_, not only in the univariate analysis but also in the multivariate analysis (Model 2: p < 0.0001, p < 0.0001, p = 0.02, and p = 0.003, respectively). However, no significant association was observed between the presence of DS and SpO_2_ <90% (Table 4). The presence of USPs was significantly associated with a higher value of SpO_2_ <90%, even after adjusting for potential confounders (p = 0.005).

**Table 4:** Factors for presence of estimated sleep-disordered breathing and hypoxemic status.

Model 1	Estimated SDB		3%ODI		Averaged SpO_2_		SpO_2_<90%		Nadir SpO_2_	
	OR (95% CI)	P-value	β	P-value	β	P-value	β	P-value	β	P-value
DS	6.24 (2.55–15.26)	<0.0001	0.47	<0.0001	−0.29	0.004	0.14	0.18	−0.32	0.001
Male	0.67 (0.30–1.49)	0.33	0.01	0.94	−0.06	0.59	0.07	0.48	0.07	0.46
Age	0.97 (0.87–1.09)	0.64	−0.04	0.66	0.06	0.59	−0.20	0.047	0.28	0.003
BMI	1.05 (0.92–1.20)	0.43	0.10	0.30	−0.06	0.54	−0.01	0.90	0.06	0.53
Unusual sleep posture	2.54 (1.09–5.95)	0.03	0.30	0.001	−0.19	0.06	0.30	0.003	−0.22	0.02
Model 2	OR (95% CI)	P-value	β	P-value	β	P-value	β	P-value	β	P-value
DS	5.98 (2.22–16.10)	<0.0001	0.42	<0.0001	−0.26	0.02	–	–	−0.30	0.003
Male	–	–	–	–	–	–	–	–	–	–
Age	–	–	–	–	–	–	−0.17	0.08	0.29	0.001
BMI	–	–	–	–	–	–	–	–	–	–
Unusual sleep posture	1.10 (0.41–2.98)	0.85	0.10	0.32	−0.06	0.59	0.28	0.005	−0.06	0.57

Model 1: Clued, Model 2: Multivariate analysis. Hyphen indicates variable which did not include the multivariate analysis.

SDB: Sleep-disordered breathing, ODI: Oxygen desaturation index, DS: Down syndrome, BMI: Body mass index.

### 3.4 SDB-related signs and symptoms and overnight pulse oximetry parameters in children with DS, with or without estimated SDB

The 51 children with DS were further divided into two groups: those with and without estimated SDB (Table 5). Between the two groups, the 3% ODI, average SpO_2_, SpO_2_ <90% ratios, and nadir SpO_2_ were significantly worse in children with DS and estimated SDB (p < 0.0001, p = 0.02, p = 0.0001, and p = 0.009, respectively). However, the presence of USPs was not significantly different between the two groups.

**Table 5:** Difference of Characteristics between DS people with estimated SDB and those without estimated SDB.

	With SDB	w/o SDB	P-value
**Number**	26	25	–
**Age**, years	8.5 (7.0–12.0)	7.0 (5.0–10.0)	0.14
**Male**, N (%)	12 (46.2)	14 (56.0)	0.48
**Physique**			
Body weight, kg	22.0 (17.5–40.0)	19.0 (15.8–24.0)	0.09
Body height, cm	110.0 (100.0–125.5)	118.5 (108.0–140.0)	0.14
BMI, kg/m^2^	16.4 (15.7–20.4)	15.7 (15.1–17.8)	0.22
**CHD**, N (%)	16 (61.5)	19 (76.0)	0.27
**Questionnaire**			
Arousal, N (%)	18 (69.2)	11 (44.0)	0.07
Snoring, N (%)	16 (61.5)	13 (54.2)^a^	0.60
Apnea, N (%)	7 (43.8)^a^	3 (15.0)^a^	0.056
Napping, N (%)	6 (24.0)^a^	10 (45.5)^a^	0.12
ESS, points	2.0 (1.0–4.0)	2.0 (1.0–5.0)	0.87
**Overnight oximetry**			
3%ODI, dips/hour	7.5 (5.9–12.9)	3.2 (2.2–4.2)	<0.0001
Averaged SpO_2_, %	96.2 ± 0.9	96.8 ± 0.6	0.02
SpO_2_<90%, %	17.0 (5.0–26.0)	3.0 (1.0–4.0)	0.0001
Nadir SpO_2_, %	80.6 ± 5.2	85.3 ± 4.2	0.0009
**Sleep postures**			
Unusual, N (%)	13 (50.0)	14 (56.0)^a^	0.67
Leaning forward	11 (42.3)	14 (56.0)^a^	0.33
Sitting	6 (24.0)^a^	3 (13.6)^a^	0.37
Prone, N (%)	7 (28.0)	6 (23.1)	0.69

DS: Down syndrome, SDB: Sleep-disordered breathing, N: Number, BMI: Body mass index, CHD: Congenital heart diseases, ESS: Epworth sleepiness scale, ODI: Oxygen desaturation index.

a Since each answer had missing data (ie, answered as “Unknown”), the denominator of the field differed from the others.

## 4 Discussion

This case–control study clarified the following: approximately 50% of children with DS had SDB, based on the index of 3% ODI ≥5 dips/h (estimated SDB), while only 14% of CC had SDB; estimated SDB tended to increase in children with DS but decreased in CC along with growth; children with DS had more severe SDB than that of CC, based on the questionnaires and overnight oximetry survey; and children with USPs, which were more frequent in DS, might experience severe hypoxemia caused by SDB.

A previous study using type-1 PSG showed that while 66.4% of children with DS had OSA [21], less than 50% of them who reported sleep problems underwent PSG [22]. Moreover, the former study reported that the prevalence of OSA was 53.8% in children with DS, even in those with a negative history of SDB [21]. These facts imply that many of the children with DS might have missed an appropriate reference timing to improve their sleep quality. The latter study also showed that younger age was associated with more severe SDB, which supported our result that younger age was associated with a higher value of SpO_2_ <90% [22]. Regarding adverse impacts of SDB on children with DS, previous studies showed that SDB degraded the levels of verbal intelligence quotient, verbal fluency, and attentional functions [9]. In addition to the association between SDB and cognitive function, lack of deep sleep (ie, sleep stage N3) in children with DS was significantly associated with lack of adaptive behavior [9]. These studies indicated that the presence of SDB could be a risk factor for neurocognitive dysfunction in children with DS. Regarding the effects of SDB on cardiovascular function, previous studies showed SDB significantly associated with high blood pressures, probably due to over-activation of sympathetic nerve activity and arterial endothelial dysfunction [8]. Moreover, a previous study showed that severe SDB in people with DS was associated with reduced cardiac function [9]. To avoid these undesirable effects of SDB on children, early detections of SDB are necessary to ensure better physical and mental development. The American Academy of Pediatrics particularly recommended screening for SDB to manage children with DS [11].

Another study using questionnaires showed that SDB-related signs and symptoms and daytime sleepiness were more frequent in children with DS than in typically developing children [23]. The multivariate analyses in this study also showed that the prevalence of observed snoring and daytime napping, including higher ESS values, was higher in children with DS than in CC. These trends were in agreement with previous reports, stating that loud snoring was common in patients with DS [23]. The parental reports of loud snoring were associated with objective measures of audio recording during sleep and with a reduction in blood oxygen levels during sleep. Other studies also showed that the presence of snoring was a strong predictor of OSA [23,24]. Some previous studies used parental responses to questions about sleep and SDB [23,25]. Moreover, a previous study reported that witnessed apnea during sleep was an independent positive predictive factor for OSA in children [26]. We also documented that apnea was witnessed in 27.8% of children with DS, while none of the CC showed apnea during sleep. The estimated SDB in pre-pubertal children (4–10 years) was higher than that in pubertal children (11–15 years) in the CC, while BMI in both groups were within normal range. One of the possible reasons might be adenotonsillar hypertrophy, which is frequently observed in children aged 2–8 years old and declines afterward [27]. In children with DS, such a decrease of estimated SDB between pre-pubertal and pubertal children was not observed, and other SDB-related parameters and USPs were consistently high in both pre-pubertal and pubertal periods. This suggested that there were different causes, not related to adenotonsillar hypertrophy. The values of BMI in the pubertal period were significantly larger in the children with DS than in CC, which might contribute to the higher frequency of SDB in pubertal periods. Other characteristics specific to DS, such as hypotonia, macroglossia, and midface hypoplasia, might also influence the increased tendency of estimated SDB.

Regarding sleeping position, children with DS frequently showed USPs regardless of the age group, but the ratio of USPs in CC decreased with growth. With increasing age, the ratio of SDB-related signs and symptoms, such as witnessed midnight arousal, apnea, and daytime sleepiness (ESS values), were more frequent in children with DS than in CC, only in the age group 11–15 years. The BMI was not significantly different between children with DS and CC in the age groups 4–5 years and 6–10 years but was higher in children with DS than in CC in the age group 11–15 years. Such differences in the BMI between children with DS and CC could lead to a higher rate of observed SDB-related signs and symptoms in children with DS in the age group 11–15 years. Prevention of obesity seems important in DS after the children's heights stop increasing. Previous studies have also shown a close relationship between USPs and SDB-related signs and symptoms, indicating that USPs might alleviate SDB in DS [10,14]. Although it would be possible that USPs alleviate SDB in DS, a significant association was observed only between USPs and accumulated hypoxemia (ie, SpO_2_ <90%) in the present study, using a multivariate analysis. USPs could have helped avoid airway collapse during sleep [15], but the frequency of USPs was not different between children with DS, with and without estimated SDB (Table 5). The exact reason for these findings were unclear, but it cannot be denied that USPs could provide some protection against SDB (ie, positional therapy) by reducing the severity of SDB in children with DS. Thus, pulse oximetry parameters should be evaluated in terms of sleeping position when the data are recorded. In our study population with DS, the real prevalence of SDB might increase after real-time recordings of both sleeping postures and oximetry parameters.

Our study showed that a high prevalence of estimated SDB, high value of 3% ODI, and low values of average SpO_2_ and nadir SpO_2_ were consistently related to the presence of DS after adjusting for several confounding factors. Overnight pulse oximetry is an easy, low-cost, and useful screening tool for SDB in children when PSG is unavailable or difficult to perform owing to intellectual disability [28]. A previous study demonstrated that home pulse oximetry screening could decrease the number of children with DS requiring multichannel sleep studies, and this might substantially reduce the burden on children, families, and health services alike [12].

### 4.1 Limitations

This study had some limitations. First, this was a cross-sectional study that did not allow for the inference of cause and effect. Second, the questionnaire used in this study adopted SDB-related signs and symptoms on the basis of findings from previous studies that have not been standardized or validated for this particular group with DS. Third, we could not perform positional monitoring during pulse oximetry, and thus, we could not assess the direct relationship between USPs and SDB. While the evaluation using PSG would allow us a detailed assessment of SDB, the sleep test using PSG would be difficult in specific types of children, such as young children with severe intellectual disabilities, like those in our study. We consider this a trade-off between a higher quality test in a limited number of patients and a comparably limited quality test in more patients. Taking this point into consideration, we decided to use an oximetry test, which might be advantageous for the young children with severe intellectual disabilities in this study. Since the criteria for evaluating the presence of SDB in children have not yet been established, we used the same criteria as those for adults [4]. This might have resulted in inappropriate diagnoses of SDB in children. The development of specific criteria of SDB for children have not yet been established. To more precisely assess the suitable cutoff value of pulse oximetry screening when evaluating the presence of SDB, future studies should investigate the cutoff value, including its sensitivity and specificity for screening, using overnight oximetry and monitoring sleeping positions. Since we recognized that the assessment of SDB using pulse oximetry had many limitations for the exact diagnoses of SDB, we think that the future studies based on PSG are mandatory to more precisely assess SDB and the relationships between USPs and SDB. Additionally, we obtained SDB-related signs and symptoms from parental replies to the questionnaires, which inevitably introduces ambiguity, although this could not be avoided, as it was unlikely for children with DS to correctly answer the questionnaire by themselves. In this regard, the use of recently developed screening questionnaires specifically for SDB in children with DS would have been valuable [29]. We could not obtain data regarding the levels of intellectual disability in conjunction with the International Classification of Diseases 10th Editions Clinical Modification (ICD-10) in the children with DS participating our study since the questionnaires were completed by their caregivers, who were non-medical personnel. We think that it would be difficult for parents to judge objective intellectual disability levels, classified by the ICD-10 code, for their children with DS. Despite these limitations, the population-based sampling frame improved the external validity of the study findings when compared with those of studies conducted only among patients with DS. The use of the Epworth Sleepiness Scale for Children and Adolescents (ESS-CHAD) might overcome this problem and be more useful for assessing excessive daytime sleepiness in this particular population [30]. However, we could not use ESS-CHAD in this study since the study began in 2015 when the Japanese version of ESS-CHAD had not yet been developed. In the parental reports of signs of daytime sleepiness, a higher rate of daytime napping (28–40%) than that of the self-reports of ESS in children with DS was observed. We believed that parental reports would be reliable compared with the results of ESS in those children since we did not use ESS-CHAD. The parental reports indicated that children with DS might frequently have sleep-related problems, such as SDB, and the problems might worsen their daytime activations. We recognized this as a limitation of the study, but parental recognition of the high rates of daytime napping in children with DS might be important to manage the children. It is well known that the presence of SDB is associated with the presence of snoring or apnea. We excluded this variable from Model 2 because of multicollinearity. Lastly, we could not confirm the reliability of overnight pulse oximetry examinations using PSG. While the evaluation using PSG enabled us to assess SDB-related data in detail, the sleep test using PSG would be difficult in some children, such as young children with severe intellectual disabilities who might be present in the group of children with DS. The difficulty of PSG would lead to a significant selection bias in this study. Thus, we used an oximetry test, which might be advantageous for young children with severe intellectual disabilities.

## 5 Conclusions

Our study showed that SDB was more prevalent and severe in children with DS than in CC, and the difference in prevalence became more obvious with age. The children with USPs, who were more frequent in cases of DS, might suffer from more severe hypoxemia due to SDB than those without USPs. More attention should also be paid to children with DS, especially those with USPs, for the early detection and treatment of SDB. This study indicated the high prevalence of SDB in children with DS compared with CC and aimed to detect SDB with the combination of overnight pulse oximetry examination and parental reports of SDB-related signs, including the USPs. This assessment might be useful for early detection of SDB, especially in children with intellectual disabilities who might not be able to participate in repetitive or formal sleep tests.

## Authorship statement

H.S., A.R., M.N., S.A., and A.C. were contributed to “Study Design”. H.S., A.R., N.M., K.F., and N.H. were contributed to “Data Collection”. H.S. and A.R. were contributed to “Analyzing data/Writing the paper”. All authors were contributed to “Interpretation of data/Revising the manuscript critically for important intellectual content”.
