# Supplementary material for: Characteristics of sleep-disordered breathing in children with down syndrome - A comparison with typically developing children
Source: Sleep Med X. 2022 Apr 7;4:100045. doi: 10.1016/j.sleepx.2022.100045 (PMC9044005; doi:10.1016/j.sleepx.2022.100045)
Supplement: Supplementary file 2 — Multimedia component 2 [file mmc2.docx]

**Supplementary Table 1**

(A) Typically developed children

|  | 4-10 years | 11-15 years | P-value |
| --- | --- | --- | --- |
| **Number** | 45 | 18 | - |
| **Age,** years | 6.3±1.7 | 13.3±1.1 | <0.0001 |
| **Male**, N (%) | 24 (53.3) | 10 (55.6) | 0.87 |
| **Physique** |  |  |  |
| Body weight, kg | 21.0 (18.0-25.0) | 45.5 (40.0-53.0) | <0.0001 |
| Body height, cm | 117.1±12.2 | 155.2±8.1 | <0.0001 |
| BMI, kg/m^2^ | 15.4 (14.6-16.9) | 18.8 (17.7-19.7) | <0.0001 |
| **CHD**, N (%) | 0 (0.0) | 0 (0/0) | - |
| **Questionnaire** |  |  |  |
| Arousal, N (%) | 15 (33.3) | 7 (38.9) | 0.68 |
| Snoring, N (%) | 13 (31.0) | 5 (33.3) | 0.87 |
| Apnea, N (%) | 0 (0.0) ‡ | 0 (0.0) ‡ | - |
| Napping, N (%) | 3 (6.8) ‡ | 3 (16.7) | 0.23 |
| ESS, points | 1.0 (0.0-1.0) | 1.0 (0.0-2.0) | 0.71 |
| Subjective sleeping time, hour | 8.8 (7.8-9.7) | 6.8 (6.2-7.4) | 0.0004 |
| **Overnight oximetry** |  |  |  |
| Estimated SDB, N (%) | 9 (20.0) | 0 (0.0) | 0.04 |
| 3%ODI, dips/hour | 1.6 (1.0-2.8) | 1.2 (0.5-2.0) | 0.09 |
| Averaged SpO_2_, % | 97.0±0.9 | 97.2±0.6 | 0.52 |
| SpO_2_<90%, % | 1.5 (0.0-4.0) | 1.0 (0.0-2.0) | 0.33 |
| Nadir SpO_2_, % | 86.9 (82.0-90.0) | 88.0 (85.8-93.0) | 0.12 |
| **Sleep postures** |  |  |  |
| Unusual, N (%) | 6 (13.3) | 0 (0.0) | 0.10 |
| Leaning forward | 6 (13.3) | 0 (0.0) | 0.10 |
| Sitting | 0 (0.0) ‡ | 0 (0.0) ‡ | - |
| Prone, N (%) | 18 (40.9) ‡ | 5 (29.4) ‡ | 0.41 |

N: Number, BMI: Body mass index, CHD: Congenital heart diseases, SDB: Sleep-disordered breathing, ESS: Epworth sleepiness scale, ODI: Oxygen desaturation index

‡: Since each answer had missing data (i.e., answered as “Unknown”), the denominator of the field differed from the others.

(B) Down syndrome

|  | 4-10 years | 11-15 years | P-value |
| --- | --- | --- | --- |
| **Number** | 35 | 16 | -- |
| **Age,** years | 6.6±1.9 | 13.0±1.2 | <0.0001 |
| **Male**, N (%) | 17 (48.6) | 9 (56.3) | 0.61 |
| **Physique** |  |  |  |
| Body weight, kg | 18.0 (15.0-20.0) | 42.8 (38.8-46.5) | <0.0001 |
| Body height, cm | 106.7±10.3 | 140.3±6.9 | <0.0001 |
| BMI, kg/m^2^ | 15.7 (14.9-16.7) | 21.0 (18.9-22.3) | <0.0001 |
| **CHD**, N (%) | 27 (77.1) | 8 (50.0) | 0.053 |
| **Questionnaire** |  |  |  |
| Arousal, N (%) | 17 (48.6) | 12 (75.0) | 0.08 |
| Snoring, N (%) | 19 (54.3) | 10 (66.7) | 0.42 |
| Apnea, N (%) | ‡5 (20.0) | ‡5 (45.5) | 0.12 |
| Napping, N (%) | ‡10 (31.3) | ‡6 (40.0) | 0.56 |
| ESS, points | 2.0 (1.0-4.0) | 2.0 (2.0-5.0) | 0.23 |
| Subjective sleeping time, hour | 9.0 (8.2-9.4) | 8.4 (7.7-9.4) | 0.20 |
| **Overnight oximetry** |  |  |  |
| Estimated SDB, N (%) | 16 (45.7) | 10 (62.5) | 0.27 |
| 3%ODI, dips/hour | 4.8 (3.1-7.6) | 5.6 (3.3-9.5) | 0.64 |
| Averaged SpO_2_, % | 96.5 (96.2-97.0) | 96.7 (96.0-97.0) | 0.80 |
| SpO_2_<90%, % | 5.0 (3.0-10.0) | 3.0 (2.0-20.0) | 0.54 |
| Nadir SpO_2_, % | 81.3 (79.0-86.0) | 85.7 (81.2-88.1) | 0.15 |
| **Sleep postures** |  |  |  |
| Unusual, N (%) | 17 (48.6) | 10 (62.5) | 0.36 |
| Leaning forward | 15 (42.9) | 10 (62.5) | 0.19 |
| Sitting | 5 (16.1) ‡ | 4 (25.0) | 0.46 |
| Prone, N (%) | 8 (22.9) | 5 (31.3) | 0.52 |

N: Number, BMI: Body mass index, CHD: Congenital heart diseases, SDB: Sleep-disordered breathing, ESS: Epworth sleepiness scale, ODI: Oxygen desaturation index

‡: Since each answer had missing data (i.e., answered as “Unknown”), the denominator of the field differed from the others.
